# Mobile App–Based Lifestyle Coaching Intervention for Patients With Nonalcoholic Fatty Liver Disease: Randomized Controlled Trial

**DOI:** 10.2196/49839

**Published:** 2024-02-15

**Authors:** Oh Young Kwon, Mi Kyung Lee, Hye Won Lee, Hyerang Kim, Jae Seung Lee, Yeonsoo Jang

**Affiliations:** 1 College of Nursing, Brain Korea 21 FOUR Project Yonsei University Seoul Republic of Korea; 2 College of Nursing, Mo-Im Kim Nursing Research Institute Yonsei University Seoul Republic of Korea; 3 Frontier Research Institute of Convergence Sports Science Yonsei University Seoul Republic of Korea; 4 Department of Internal Medicine, College of Medicine Yonsei University Seoul Republic of Korea; 5 Yonsei Liver Center, Severance Hospital Seoul Republic of Korea; 6 Department of Nursing Science VISION College of Jeonju Jeollabuk-Do Republic of Korea

**Keywords:** lifestyle modification, mobile health, nonalcoholic fatty liver disease, self-management, randomized controlled trial

## Abstract

**Background:**

Lifestyle modification in patients with nonalcoholic fatty liver disease (NAFLD) is key to improving health outcomes. Mobile health technologies may offer potential effective and efficient health care support to facilitate self-management.

**Objective:**

This study aims to develop a lifestyle coaching intervention using a mobile app for patients with NAFLD and evaluate physiological and psychological health outcomes for 6 months.

**Methods:**

This study was a randomized controlled trial. The personalized lifestyle coaching intervention using a mobile app was developed through established guidelines and literature reviews. This intervention consisted of information on NAFLD management, diet and physical activity self-monitoring, and coaching sessions based on patient records and SMS text messages. A total of 102 individuals were enrolled in the study and randomly assigned to the intervention group (n=48) or the control group (n=54). The outcomes were improvements in physiological (weight, liver fat score, aspartate aminotransferase, alanine transferase, and gamma-glutamyl transferase) and clinical outcomes (self-management, NAFLD self-management knowledge, self-efficacy, fatigue, depression, and quality of life). Data were analyzed using descriptive analysis and a linear mixed model to test the effects of the intervention.

**Results:**

All participants completed the study. The mean age of the participants was 48.9 (SD 13.74) years, 38.2% (39/102) were female participants, and 65.7% (67/102) were married. There were no differences in baseline demographic and clinical data between the intervention and control groups. Changes from baseline to 6 months were significant only within the intervention group for weight (*P*<.001), liver fat score (*P*=.01), aspartate aminotransferase (*P*=.03), alanine transferase (*P*=.002), gamma-glutamyl transferase (*P*=.04), self-management (*P*<.001), fatigue (*P*=.005), depression (*P*=.003), and quality of life (*P*<.001). The differences between the 2 groups for the changes over the 6 months were significant in self-management (*P*=.004), self-management knowledge (*P*=.04), fatigue (*P*=.004), depression (*P*=.04), and quality of life (*P*=.01). However, the intervention-by-time interaction was significantly effective only for self-management (*P*=.006) and fatigue (*P*=.02).

**Conclusions:**

Nonpharmacological interventions using a mobile app may be effective in improving the physiological and psychological health outcomes of patients with NAFLD.

**Trial Registration:**

Clinical Research Information Service KCT0005549; http://tinyurl.com/y2zb6usy

## Introduction

Nonalcoholic fatty liver disease (NAFLD) is the most common liver disease globally, and its incidence continues to increase rapidly. Recently, the prevalence of NAFLD worldwide was estimated to be around 32.4%, and its reported overall incidence was estimated to be 46.9 cases per 1000 persons every year [1]. Its prevalence in Asian countries ranges from 5% to 30% and is reported to have increased by 30.3% over the last 10 years in South Korea [2].

Most patients with NAFLD are asymptomatic, but more than half may develop nonalcoholic steatohepatitis, which can progress to liver fibrosis, cirrhosis, and liver cancer [1,3]. Moreover, early NAFLD is often associated with metabolic syndromes, obesity, and diabetes [4]. The progressive nature of NAFLD and the association of these factors may put a strain on health care systems in the future if not addressed in advance. Therefore, the early diagnosis and management of NAFLD have the potential to improve the health outcomes of the patients.

Lifestyle modification is key to improving the histologic condition of NAFLD following weight loss. Clinical practice guidelines recommend a weight loss of at least 5% in patients with NAFLD who are overweight or obese [5]. Previous studies have reported the effects of regular exercise practice, associated with various types of diets or not, on weight loss [6-8]. However, some factors related to long-term weight loss maintenance and motivation for weight loss remain unclear in clinical practice [9-11].

Mobile health technology has contributed to improving people’s health conditions. Apps on mobile phones can deliver health care information beyond clinical settings and enable tailored interventions to facilitate healthy lifestyles [12,13]. Furthermore, because of the possibility of real-time interactions between people without restrictions on time and place, such applications are effective in helping patients achieve specific health goals through continuous and comprehensive information delivery through SMS text messages, informative texts, illustrations, and instructional videos [14,15]. Given these benefits, mobile apps have been used to manage various diseases daily and are expected to help patients overcome obstacles in the management of NAFLD. Lifestyle modification interventions using mobile apps are increasingly being implemented for patients with NAFLD. A recent meta-analysis reported that interventions using eHealth technologies were effective for BMI, aspartate aminotransferase (AST), and alanine transferase (ALT) in these patients [16]. However, previous studies have focused on physiological outcomes related to weight, BMI, and clinical findings rather than psychological aspects. Some studies have found associations between NAFLD (with or without fibrosis) and mental health status, such as depressive symptoms, anxiety, and general quality of life decline [17-19]. However, studies that focus specifically on the psychological and physical health aspects of patients with NAFLD are lacking.

This study aimed to evaluate the effects of a lifestyle coaching intervention on self-management behaviors and the treatment of patients with NAFLD. We developed a long-term lifestyle coaching intervention routine through a mobile app aiming to improve the health of patients with NAFLD. We hypothesized that the use of this app for 6 months would be associated with improvement of NAFLD self-management compared to traditional follow-up care and would be effective in improving physiological and psychological health parameters.

## Methods

### Study Design

A randomized controlled trial was conducted using parallel treatment groups for 6 months. Participants were randomly allocated to either the self-management app for repeat tutoring (SMART-Liver, intervention group) or the standard care group (control group) in a 1:1 ratio.

### Study Participants and Setting

The participants were recruited from the outpatient department of gastroenterology at a tertiary university hospital in Seoul, South Korea. In total, 138 patients with NAFLD were assessed for eligibility and participated in a study between November 2020 and January 2022. Inclusion criteria were patients who were aged 19 years or older, diagnosed with NAFLD without secondary causes for liver fat accumulation, able to read and write in English, and owned a smartphone using an Android system. The exclusion criteria were a history of alcohol intake of more than 20 g per day for women and 30 g per day for men based on the guidelines of the Korean Association for the Study of the Liver [20], those with hepatitis B or C virus infections, pregnant women who received hepatotoxic medication, patients who had cirrhosis, those with end-stage disease, those with concomitant liver disease, those diagnosed with psychiatric conditions, and those with clinically or biochemically recognized systemic diseases. The patients were screened for eligibility by clinicians and researchers.

### Sample Size

Power analysis was performed using G*Power software (version 3.1; Heinrich Heine University) to determine the sample size. The sample size for each group was calculated to be at least 64 participants to obtain a 0.5 effect size, α=.05, and 80% statistical power. Accounting for a 10% dropout rate, the total sample size was planned to be 140 participants.

### Randomization and Allocation

The recruited study participants were patients whose physicians recommended screening for eligibility when they visited the clinic. Participants who agreed to participate in the study and provided written informed consent were randomly assigned to either the control or intervention group in a 1:1 ratio. The allocation sequence was concealed using lots marked with the letter I (intervention group) or C (control group) in sealed, impenetrable envelopes prepared by a research assistant who was not involved in the intervention. The lots were stored in cabinets with locks before and after recruitment.

Participants in the intervention group received a unique code number for logging in to the SMART-Liver app after learning how to use the app and registering it on the spot. We provided a written manual for the SMART-Liver app to each participant in the intervention group. The research assistant surveyed participants in the intervention and control group using a questionnaire to obtain their general characteristics, self-management levels, and other psychosocial behaviors. The study period of 24 weeks began after a week in both groups. Participants in the control group received routine care based on treatment protocol in the hospital.

### Intervention

#### Development of SMART-Liver Program

**Figure 1:**
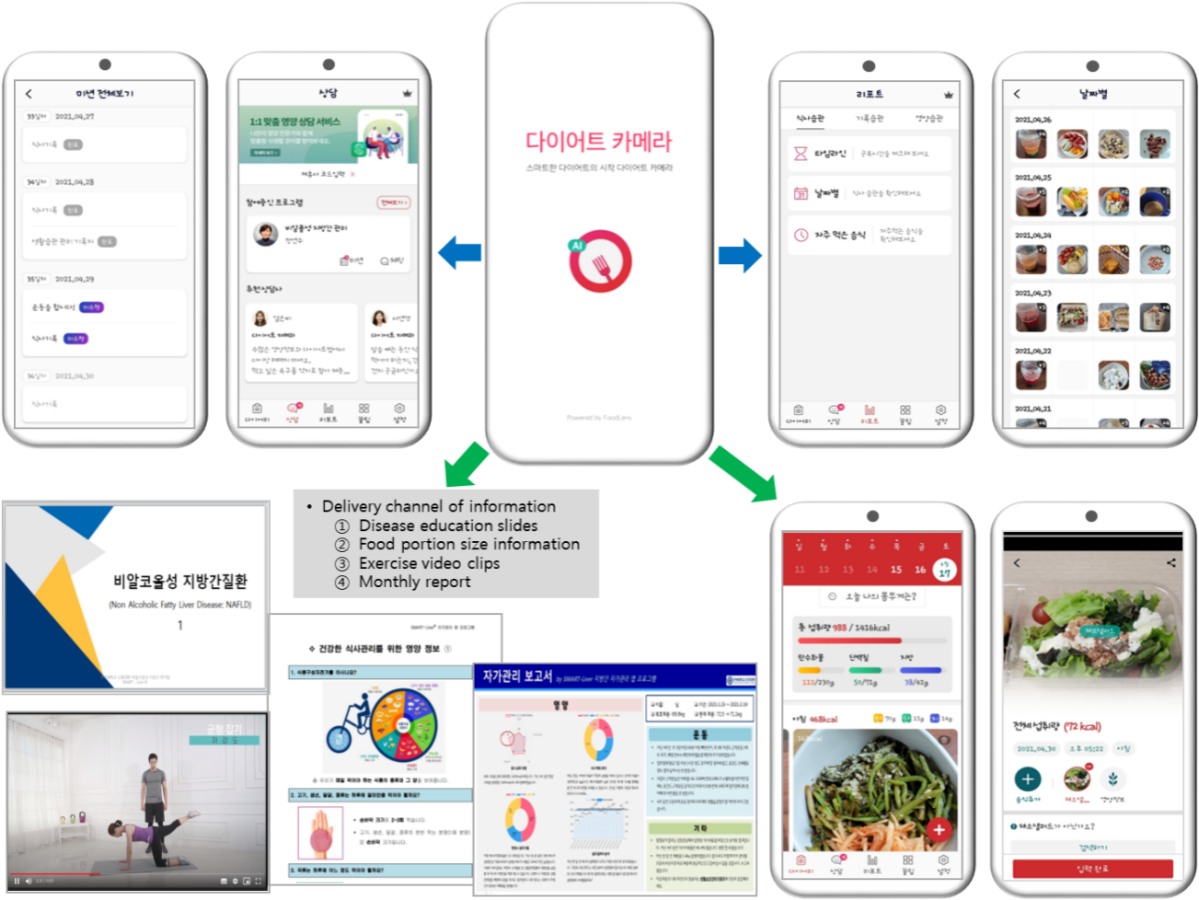
SMART-Liver app.

The SMART-Liver (self-management app for repeat tutoring) program is a lifestyle coaching intervention designed to improve the self-management of patients with NAFLD. The intervention was developed following the process of the ADDIE (Analysis, Design, Development, Implementation, and Evaluation) model [21]. The needs of patients with NAFLD and existing studies using mobile apps for self-management behaviors were analyzed in the analysis step, which included literature reviews and the guidelines for NAFLD treatment. Aiming to improve the level of self-management of patients with NAFLD, the intervention consisted of modules for self-monitoring, information, coaching, and SMS text messages related to diet and physical activities. An app with artificial intelligence technology was selected to operate the functions of self-monitoring of diet, which enabled participants to log into the app their diet at every meal. The app was created by modifying the Diet Camera app developed by Doing Lab in South Korea and is commercially available in app stores with basic features that are accessible for free. Full features of the app were made available to participants for the SMART-Liver intervention in collaboration with Doing Lab during the intervention period. A representation of the Smart-Liver app interface is shown in Figure 1.

The information provided in the app include (1) educational slides for NAFLD treatment based on guidelines and diet tips, (2) diet news for 1 portion size of food, (3) exercise video clips, and (4) summary quizzes to facilitate self-management behaviors.

Self-monitoring instructions include (1) diets for every meal and snack; (2) checking of the total caloric intake based on their records; and (3) lifestyle health habits such as exercise, sleep, alcohol intake, and smoking.

The participants were coached to (1) meet with the research team and physical activity specialist to set personal weight and physical activity goals based on their weight and diet information logged in the first week after registering in the app, (2) identify barriers and facilitators in their performances of health-related behavioral change, (3) evaluate or reset the goal in the 12th week, and (4) provide feedback through reports that displayed analyses of their diet logs every 4 weeks for 6 months.

The participants received (1) SMS text messages automatically every morning at 8 AM to remind participants about daily missions, including diet logs; (2) SMS text messages when the missions were completed to encourage the participants’ performances; (3) tailored feedback messages for diet and physical activity records once a week; and (4) real-time messages using a chat channel when they have any queries about their disease or obstacles encountered.

#### SMART-Liver Intervention

The participants input their body weight and height in the app and received information on the proper amount of calories for their weight through a chat channel every week in the first month. During the rest of the intervention period, the researchers continuously reminded the participants to incorporate this information into their daily routines. From the day of registration onward, participants logged their diets, such as the type of food and the amount they ate, and answered questions about lifestyle management related to exercise, smoking habits, alcohol consumption, and sleep patterns. Participants could check or edit their logs in the app. After using the app for 1 week to practice, they set a weight loss goal based on their records of diet, exercise, and lifestyle logged into the app, with the assistance of a dietitian and a physical activity specialist via remote meeting. After this meeting, the actual intervention program was initiated. Daily missions such as diet logs or weight measurements were sent every morning at 8 AM. During the first month, individually catered educational exercise video clips and information materials on liver disease and diet were sent weekly. After the first month, participants continued to have access to these educational materials on the app at any time.

In addition, the research team sent feedback messages based on the performance rate of diet records every week to encourage compliance and provided monthly feedback reports that included comments from dietitians and physical activity specialists via the app’s chat channel. If there were any questions or obstacles to participating in the intervention, participants could ask the research team using a chat channel, and the research team responded in real time during working hours announced in advance. In the third month, participants reset their goals for body weight or lifestyle health habits for the remaining 3-month period through counseling with a dietitian and physical activity specialist.

In the control group, participants received phone calls from the research team a week after enrollment. The research team took note of the participants’ body weights and provided information on the importance of weight loss, diet, and exercise for NAFLD management via phone calls. The participants received the standard care for NAFLD for 6 months and were reached by phone calls in the third and sixth months for weight measurement and survey.

### Measurements

#### Overview

We measured general characteristics, physiological, and clinical outcomes over 6 months. Clinical outcomes were measured at baseline and 3 and 6 months. Clinical data were obtained at baseline and after 6 months.

#### General and Clinical Characteristics

Demographic and clinical information including age, sex, marital status, education level, family income, health-related factors, comorbidities, and clinical findings (liver fat score, AST, ALT, and gamma-glutamyl transferase [ɣ-GT]) were collected at the beginning of the study via survey.

#### Self-Management

Self-management was measured using the NAFLD Self-Management Questionnaire developed based on the Individual and Family Self-Management Theory in patients with NAFLD in South Korea [22]. This 22-item self-report questionnaire is scored on a 5-point Likert scale ranging from 1 (never) to 5 (always). It consists of 6 subdomains: medical treatment compliance, management of medications and dietary supplements, alcohol consumption management, sleep management, family support, and lifestyle management. The mean scores were calculated, with a higher score indicating a higher level of self-management. The Cronbach α value was .87 in the original study and .84 in this study.

#### Self-Efficacy

Self-efficacy was assessed using the 22-item Chronic Disease Self-Efficacy Scale–Korean Version [23], which was modified from the Chronic Disease Self-Efficacy Scale developed by Lorig et al [24]. Responses were provided on a 10-point Likert scale ranging from 1 (not at all confident) to 10 (totally confident), with higher scores indicating higher confidence. In the original study, the Cronbach α of the scale was .91 and .93 in the Korean version. In this study, the Cronbach α was .95.

#### Fatigue

Fatigue was assessed using the Korean version of the Revised Piper Fatigue Scale [25], which was translated from the Revised Piper Fatigue Scale [26]. This scale consists of 22 items, each rated on a 10-point numeric rating scale ranging from 0 (none) to 10 (maximum symptom intensity). The scores for all items were added, and the total scores were divided by the number of items. This scale provides an estimate of fatigue, with 0 representing none, 1-3 as mild, 4-6 as moderate, and 7-10 as severe fatigue. Higher mean scores indicated higher perceived fatigue levels. The Cronbach α of the original scale was .97, and that of the Korean version was .96. In this study, the Cronbach α was .97.

#### Depression

Depression was assessed using the Beck Depression Inventory I-Korean version [27], which is composed of 21 items. Each item is rated on a 4-point Likert scale ranging from 0 to 3. The total score ranges from 0 to 63. Individuals with a total score between 0 and 13 were considered to have minimal depressive symptoms, 14-19 mild depressive symptoms, 20-28 moderate depressive symptoms, and 29-63 severe depressive symptoms. The Cronbach α of the scale was reported as .85 in a previous study and was .91 in this study.

#### Quality of Life

Quality of life was assessed using the World Health Organization Quality of Life–Brief Version [28], a self-reported questionnaire containing 26 items. The responses were rated on a 5-point Likert scale, where 1 indicated high satisfaction and 5 indicated low satisfaction. The mean score of the items was used in the analyses, and higher scores indicated a better quality of life. The Cronbach α of the scale was reported as .90 in the original study and .89 in this study.

#### Self-Management Knowledge

The participants’ self-management knowledge was evaluated using 10 items of essential knowledge in the NAFLD self-management questionnaire developed by the researchers. The items were based on the NAFLD management guidelines, and the participants answered “yes=1” or “no=0.” The total scores were added, and a higher score indicated a higher level of NAFLD self-management knowledge.

#### SMART-Liver App Compliance

Patient compliance was defined as the participants’ diet-log record rate in this study. All participants in the intervention group were expected to log their diet information 3 times using the SMART-Liver app, and their compliance with the intervention was evaluated based on diet-log rates during the 6 months. The diet logs were collected from the app and calculated by the researchers. The levels of compliance were analyzed as >70%, 70%-90%, and more than 90%.

### Data Analysis

Data analyses were performed using SPSS Statistics for Windows (version 25.0; IBM). A per-protocol analysis was conducted to evaluate the results of the intervention among 102 participants. General characteristic data are described using mean and SD or frequencies. Differences between the intervention and control groups in sociodemographic, physiological, and psychosocial variables at baseline were analyzed using a 2-tailed *t* test. This process allowed us to confirm the homogeneity between the intervention and control groups and ensured that we did not require further adjustments for any imbalance.

We conducted a paired 2-tailed *t* test for continuous variables within each group and an independent 2-tailed *t* test between groups to compare the baseline and 3- and 6-month changes. Subsequently, a linear mixed model was used to evaluate any statistically significant differences over time in repeated measurements between the study groups. This approach assessed the intervention effect by simultaneously adjusting for the correlation between the intervention and control groups and between repeated measurements of the same participants.

### Ethical Considerations

This study was approved by the institutional review board of Yonsei University Health System (IRB 4-2020-0955). All participants were provided written informed consent prior to participating. This study only recruited participants who were willing to participate, and they were fully informed of the rights and obligations associated with the intervention. They retained the freedom to withdraw from the study at any time. All study-related and participant data were securely stored in a password-protected file cabinet with restricted access. To ensure the confidentiality of participants, all identifiable data were de-identified using coding. Participants were compensated for their participation and completion of the study survey on three occasions (Baseline, 3 months, and 6 months). At the 3-month survey, participants received US $15 upon completion of both the baseline and 3-month surveys. Additionally, participants who completed the 6-month intervention were provided with a free transient elastography test for liver fat valued at US $150.

The trial was prospectively registered in the World Health Organization–accredited Clinical Research Information Service on October 29, 2020 (registration number KCT0005549). We performed this trial based on the CONSORT-EHEALTH (Consolidated Standards of Reporting Trials of Electronic and Mobile Health Application and online TeleHealth) guidelines (Multimedia Appendix 1).

## Results

### Characteristics of Participants

**Figure 2:**
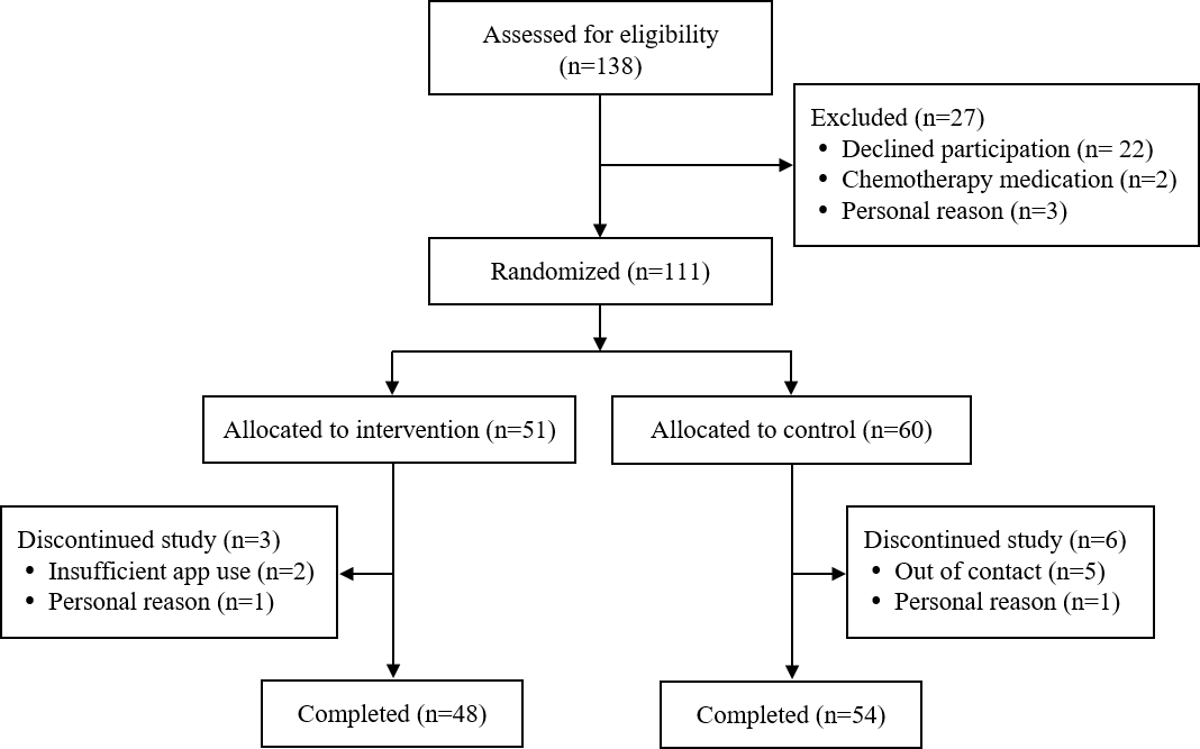
Summary of participation flow diagram.

In total, 111 participants were enrolled in this study (Figure 2). During the first month, 2 patients withdrew from the study for personal reasons. Five patients in the control group dropped out of the study because they did not respond to the several contact attempts made by the researchers. Two patients in the intervention group dropped out due to poor compliance and reluctance to use the app. Thus, a total of 102 (91.9%) participants were included in the final analysis (48 in the intervention group and 54 in the control group). Overall, the mean age of the participants was 48.9 (SD 13.74) years.

Table 1 shows the demographic data and baseline characteristics of the participants in each group. There were no significant differences between the groups in terms of age, sex, socioeconomic status, and the presence of relevant comorbidities (all *P*>.05). The physiological and psychosocial outcome variables showed no differences between the participants in either group at the beginning of the intervention (Table 2).

**Table 1:** Baseline characteristics of the intervention and control groups (N=102).

Characteristics		Control (n=54)	Intervention (n=48)	*P* value	
Age (years), mean (SD)		47.1 (13.91)	51.0 (13.37)	.17	
Sex (male), n (%)		29 (54)	34 (71)	.10	
Occupation (yes), n (%)		41 (76)	36 (75)	≥.99	
**Marital status, n (%)**					.18
	Married	36 (67)	31 (65)		
	Single	17 (32)	12 (25)		
	Widowed, divorce, or separation	1 (2)	5 (10)		
**Education, n (%)**					.12
	Elementary school	0 (0)	1 (2)		
	Middle school	0 (0)	0 (0)		
	High	22 (41)	11 (23)		
	College	23 (28)	29 (60)		
	Graduate school or over	9 (17)	7 (15)		
**Family income (US $ per month), n (%)**					.95
	≤1750	7 (13)	6 (13)		
	1751-3500	13 (24)	13 (27)		
	3501-5250	19 (36)	14 (29)		
	≥5251	14 (26)	15 (31)		
**Smoking, n (%)**					.88
	Yes	6 (11)	7 (15)		
	No	34 (63)	28 (58)		
	Quit	14 (26)	13 (27)		
Drinking (yes), n (%)		20 (37)	15 (31)	.68	
Sleep sufficiency (yes), n (%)		26 (48)	20 (42)	.55	
**Comorbidity^a^ (yes), n (%)**		41 (76)	40 (83)	.46	
	Hyperlipidemia	26 (48)	22 (46)	.85	
	Hypertension	22 (41)	18 (38)	.84	
	Diabetes	14 (26)	17 (35)	.39	
	Osteoporosis	1 (2)	1 (2)	≥.99	
	Others	20 (37)	20 (42)	.69	
**Medication^a^ (yes), n (%)**		40 (74)	42 (88)	.13	
	Hyperlipidemia	25 (46)	23 (48)	≥.99	
	Hypertension	19 (40)	19 (35)	.69	
	Diabetes	14 (26)	16 (33)	.52	
	Others	22 (41)	21 (44)	.84	

^a^Respondents are allowed to select more than 1 answer for a single question.

**Table 2:** Differences of outcome variables between 2 groups at baseline (N=102).

Variable		Control (n=54), mean (SD)	Intervention (n=48), mean (SD)	*P* value
**Physiological**				
	Weight (kg)	84.3 (15.93)	83.4 (19.21)	.81
	BMI (kg/m^2^)	29.9 (4.13)	29.6 (4.57)	.70
	Liver fat score	333.6 (97.23)	317.0 (35.15)	.27
	AST^a^ (IU/L)	36.4 (24.40)	33.5 (14.23)	.47
	ALT^b^ (IU/L)	43.3 (31.50)	42.8 (29.80)	.93
	ɣ-GT^c^	46.9 (34.86)	51.2 (40.49)	.57
**Psychosocial**				
	Self-management	3.5 (0.65)	3.5 (0.52)	.75
	Knowledge	7.6 (1.50)	7.3 (1.90)	.37
	Self-efficacy	7.2 (1.42)	7.2 (1.14)	.81
	Fatigue	4.3 (2.09)	4.3 (1.97)	.98
	Depression	8.5 (8.73)	8.6 (7.65)	.96
	Quality of life	92.2 (16.51)	91.3 (16.49)	.78

^a^AST: aspartate aminotransferase.

^b^ALT: alanine transaminase.

^c^ɣ-GT: gamma-glutamyl transferase.

### Within-Group Effects

#### Changes in Physiological Outcomes

There were significant decreases in weight and BMI from baseline to 6 months after using the app (Table 3). Weight decreased significantly in the intervention group (*P*<.001), and BMI was improved over 6 months (*P*<.001). Furthermore, there were significant reductions in the liver fat score (*P*=.01), AST (*P=*.03), ALT (*P*=.002), and ɣ-GT (*P=*.04) in participants of the intervention group. However, there were no significant improvements in the physiological factors of the control group (all *P*>.05).

**Table 3:** The changes from baseline to 6 months within each group (N=102).

Variable			Control group (n=54)							Intervention group (n=48)				
			Baseline, mean (SD)		6 months, mean (SD)		*P* value		Baseline, mean (SD)			6 months, mean (SD)		*P* value
**Physiological**														
	Weight (kg)	84.3 (15.93)		82.2 (16.10)		.13		83.4 (19.21)			80.9 (19.62)		<.001	
	BMI (kg/m^2^)	29.9 (4.13)		29.6 (4.30)		.08		29.6 (4.57)			29.0 (4.81)		<.001	
	Liver fat score	333.6 (97.23)		318.7 (35.52)		.26		317.0 (35.5)			305.4 (45.10)		.01	
	AST^a,b^ (IU/L)	37.1 (25.49)		36.2 (30.84)		.79		33.6 (14.13)			29.5 (12.52)		.03	
	ALT^c,d^ (IU/L)	42.1 (31.06)		36.5 (32.53)		.15		39.1 (24.50)			31.0 (23.34)		.002	
	ɣ-GT^e,f^	47.9 (37.64)		43.9 (33.48)		.30		50.6 (38.71)			38.4 (18.34)		.04	
**Psychosocial**														
	Self-management	3.5 (0.65)		3.6 (0.62)		.66		3.5 (0.52)			3.9 (0.66)		<.001	
	Knowledge	7.6 (1.50)		7.2 (1.28)		.11		7.3 (1.90)			7.6 (1.60)		.18	
	Self-efficacy	7.2 (1.42)		7.3 (1.58)		.80		7.2 (1.14)			7.5 (1.32)		.07	
	Fatigue	4.3 (2.09)		4.5 (2.04)		.25		4.3 (1.97)			3.7 (1.80)		.005	
	Depression	8.5 (8.73)		8.5 (8.59)		.97		8.6 (7.65)			6.8 (7.57)		.003	
	Quality of life	92.2 (16.51)		93.4 (17.56)		.35		91.3 (16.49)			98.0 (17.15)		<.001	

^a^AST: aspartate aminotransferase.

^b^Samples for the analysis in the control and intervention group were 49 and 43, respectively.

^c^ALT: alanine transaminase.

^d^Samples for the analysis in the control and intervention group were 49 and 43, respectively.

^e^ɣ-GT: gamma-glutamyl transferase.

^f^Samples for the analysis in the control and intervention group were 45 and 38, respectively.

#### Changes in Psychosocial Outcomes

In the intervention group, there were significant improvements in self-management (*P*<.001), fatigue (*P*=.005), depression (*P*=.003), and quality of life (*P*<.001) scores. However, no significant changes were observed in the control group (Table 3).

#### Between-Group Effects

There were changes in the physical and physiological outcomes in both groups, but no significant differences were observed between the intervention and control groups at 3 or 6 months (Table 4). In psychosocial outcomes, self-management was significantly improved in the intervention group at 3 months and 6 months compared to the control group (mean 0.30, SD 0.47 vs mean 0.09, SD 0.47; *P*=.02; and mean 0.37, SD 0.61 vs mean 0.03, SD 0.55; *P*=.004). Self-management knowledge also was significantly improved in the intervention group at 3 months and 6 months compared to the control group (mean 0.50, SD 1.38 vs mean –0.31, SD 1.68; *P*=.009; and mean 0.37, SD 0.61 vs mean –0.35, SD 1.59; *P*=.04). Fatigue and depression were significantly reduced in the intervention group at 6 months compared to the control group (mean –0.60, SD 1.42 vs mean 0.22, SD 1.39; *P*=.004; and mean –1.75, SD 3.84 vs mean –0.08, SD 4.05; *P*=.04). Quality of life in the intervention group had significantly improved at 6 months than that in the control group (mean 6.71, SD 12.21 vs mean 1.24, SD 9.70; *P*=.01). The treatment-by-time interaction was significant in self-management and fatigue, indicating that the intervention effects over time in these variables differed significantly between the intervention group and control group across the 6 months.

**Table 4:** The effects of intervention between the control and intervention groups (N=102).

Variable		Change from baseline, mean (SD)			Between-groups difference, mean (95% CI)		*P* value	
		Control (n=54)	Intervention (n=48)			Intervention vs control		Treatment×time
**Weight (kg)**								
	3 months	–1.57 (2.98)	–2.07 (3.11)	0.50 (–0.70 to 1.69)		.41		.65
	6 months	–0.79 (3.76)	–1.54 (2.74)	0.74 (–0.56 to 2.05)		.26		.59
**Weight loss (%)**								
	3 months	–1.82 (3.40)	–2.52 (3.51)	0.70 (–0.66 to 2.06)		.31		.32
	6 months	–1.07 (3.34)	–2.08 (3.44)	1.01 (–0.54 to 2.55)		.20		.15
**BMI (kg/m^2^)**								
	3 months	–0.57 (1.05)	–0.75 (1.09)	0.18 (–0.24 to 0.60)		.40		.47
	6 months	–0.32 (1.32)	–0.58 (0.98)	0.26 (–0.20 to 0.72)		.27		.41
**Liver fat score**								
	6 months	–14.91 (96.31)	–11.60 (30.34)	–3.30 (–32.09 to 25.48)		.82		N/A^a^
**AST^b,c,d^ (IU/L) (n=92)**								
	6 months	–0.90 (23.52)	–4.09 (12.21)	3.20 (–4.73 to 11.12)		.43		N/A
**ALT^c,e,f^ (IU/L) (n=92)**								
	6 months	–5.73 (27.04)	–8.05 (15.96)	2.32 (–6.84 to 11.48)		.62		N/A
ɣ**-GT^c,g,h^ (n=83)**								
	6 months	–3.96 (25.07)	–12.16 (34.36)	8.20 (–4.81 to 21.21)		.21		N/A
**Self-management**								
	3 months	0.09 (0.47)	0.30 (0.47)	–0.21 (–0.40 to –0.03)		.025		.12
	6 months	0.03 (0.55)	0.37 (0.61)	–0.34 0.57 to –0.11)		.004		.006
**Knowledge**								
	3 months	–0.31 (1.68)	0.50 (1.38)	–0.81 (–1.42 to –0.21)		.009		.07
	6 months	–0.35 (1.59)	0.29 (1.47)	–0.64 (–1.25 to –0.04)		.04		.23
**Self-efficacy**								
	3 months	–0.10 (0.93)	–0.03 (1.12)	–0.07 (–0.47 to 0.33)		.73		.97
	6 months	0.04 (1.03)	0.29 (1.10)	–0.26 (–0.67 to 0.16)		.230		.45
**Fatigue**								
	3 months	0.13 (1.24)	–0.13 (1.36)	0.27 (–0.24 to 0.78)		.30		.46
	6 months	0.22 (1.39)	–0.60 (1.42)	0.82 (0.27 to 1.37)		.004		.02
**Depression**								
	3 months	–0.64 (4.14)	–1.00 (3.73)	0.36 (–1.19 to 1.92)		.65		.85
	6 months	–0.08 (4.05)	–1.75 (3.84)	1.67 (0.11 to 3.22)		.04		.26
**Quality of life**								
	3 months	1.17 (8.01)	3.71 (8.11)	–2.54 (–5.71 to 0.63)		.12		.59
	6 months	1.24 (9.70)	6.71 (12.21)	–5.47 (–9.78 to –1.16)		.01		.13

^a^N/A: not applicable.

^b^AST: aspartate aminotransferase.

^c^Total numbers were not 102 because of missing data.

^d^Samples for the analysis in the control and intervention group were 49 and 43, respectively.

^e^ALT: alanine transaminase. Samples for the analysis in the control and intervention group were 49 and 43, respectively.

^f^Samples for the analysis in the control and intervention group were 49 and 43, respectively.

^g^ɣ-GT: gamma-glutamyl transferase.

^h^Samples for the analysis in the control and intervention group were 45 and 38, respectively.

#### SMART-Liver App Compliance

**Figure 3:**
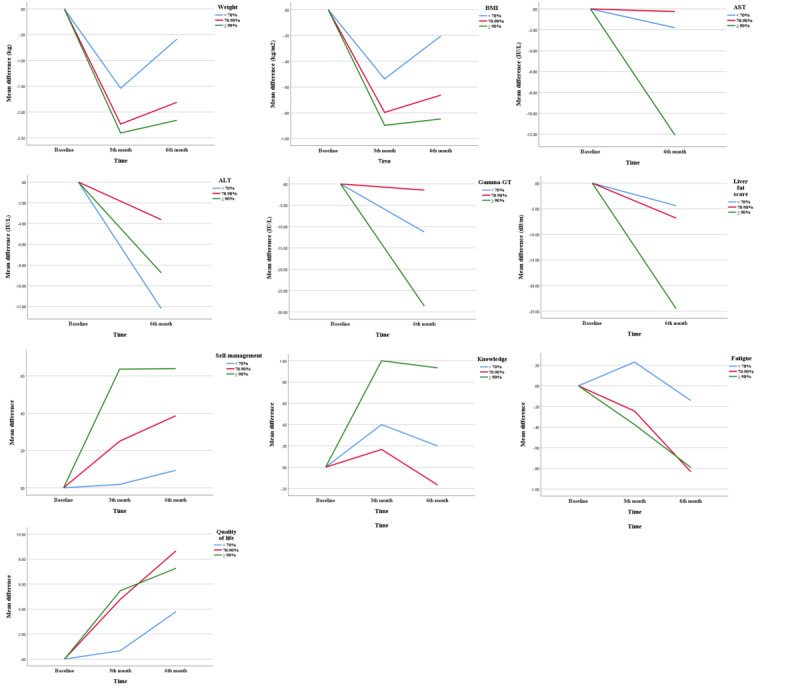
Pattern of mean changes in outcomes for 6 months in the intervention group. For a higher-resolution version of this figure, see Multimedia Appendix 2.

Mean compliance was 82.6% (SD 12.9%) at 3 months and 79.8% (SD 13.9%) at 6 months. Table 5 shows whether there were significant differences in study outcomes among 3 compliance groups in the intervention group, which were classified as 70% or less, 70% to 90%, and 90% or over. In the group with 90% or more, there were significantly greater improvements in weight loss (*P*=.01), BMI (*P*=.005), liver fat score (*P*=.002), AST (*P*<.001), and ɣ-GT (*P*=.001) at 6 months than in the groups with 70% or less and 70% to 90%. Self-management and knowledge at 3 and 6 months showed significantly greater improvement in the group with higher compliance as well (*P*<.001 and *P*<.001; *P*=.008 and *P*=.001, respectively). Fatigue in the group with 70% to 90% at 6 months was significantly lower compared with the group with 70% or less compliance (*P*=.03), and depression in the groups with 70% to 90% and with 90% or more at 3 months was higher than in the group with 70% or less (*P*=.008). The patterns of mean changes in outcomes from baseline to 6 months in each compliance group are shown in Figure 3.

**Table 5:** Changes of outcomes from baseline by the compliance level in the intervention group.

Outcome			Compliance (n=48), mean (SD)							*P* value
			A: < 70% (n=15)		B: 70%-90% (n=18)		C: ≥90% (n=15)			
**Weight (kg)**										
	3 months	–1.5 (3.97)		–2.2 (3.27)		–2.4 (1.80)		.37		
	6 months	–0.6 (2.22)		–1.8 (2.91)		–2.2 (2.92)		.01 (A<C)		
**BMI (kg/m^2^)**										
	3 months	–0.5 (1.37)		–0.8 (1.13)		–0.9 (0.69)		.26		
	6 months	–0.2 (0.77)		–0.7 (1.05)		–0.8 (1.02)		.005 (A<C)		
**Liver fat score**										
	6 months	–4.4 (23.90)		–6.8 (30.09)		–24.5 (34.00)		.002 (A=B<C)		
**AST^a^ (IU/L)**										
	6 months	–1.8 (6.35)		–0.3 (12.95)		–12.1 (13.81)		<.001 (A=B<C)		
**ALT^b^ (IU/L)**										
	6 months	–12.2 (12.36)		–3.6 (15.75)		–8.8 (19.68)		.03 (A>B)		
ɣ**-GT^c^**										
	6 months	–11.3 (21.89)		–1.4 (15.47)		–28.6 (55.21)		.001 (B<C)		
**Self-management**										
	3 months	<0.1 (0.31)		0.3 (0.48)		0.6 (0.38)		<.001 (A<B<C)		
	6 months	<0.1 (0.58)		0.4 (0.63)		0.6 (0.53)		<.001 (A<B<C)		
**Knowledge**										
	3 months	0.4 (1.30)		0.2 (1.42)		1.0 (1.36)		.008 (B<C)		
	6 months	0.2 (1.70)		–0.2 (1.50)		0.9 (0.96)		.001 (A=B<C)		
**Self-efficacy**										
	3 months	<0.1 (0.79)		–0.2 (1.28)		0.1 (1.25)		.35		
	6 months	0.4 (0.83)		0.3 (1.06)		0.2 (1.41)		.73		
**Fatigue**										
	3 months	0.2 (1.57)		–0.2 (1.20)		–0.4 (1.35)		.08		
	6 months	–0.1 (1.33)		–0.8 (1.39)		–0.8 (1.51)		.03 (A<B=C)		
**Depression**										
	3 months	–0.2 (3.88)		–1.3 (3.53)		–1.4 (3.94)		.22		
	6 months	–0.9 (2.59)		–1.8 (5.01)		–2.6 (3.27)		.10		
**Quality of life**										
	3 months	0.7 (6.81)		4.8 (8.77)		5.5 (8.15)		.008 (A<B=C)		
	6 months	3.8 (7.63)		8.7 (15.12)		7.3 (12.26)		.13		

^a^AST: aspartate aminotransferase.

^b^ALT: alanine transaminase.

^c^ɣ-GT: gamma*-*glutamyl transferase*.*

## Discussion

### Principal Findings

The purposes of this study were to develop and evaluate the physiological and psychosocial effects of the lifestyle coaching intervention for patients with NAFLD. The results showed that there were significant changes in physiological outcomes (body weight, BMI, liver fat score, AST, ALT, and ɣ-GT) and psychosocial outcomes (self-management, fatigue, depression, and quality of life) in the intervention group for 6 months, and the intervention-by-time interaction was effective for self-management and fatigue improvement.

There was a significant reduction in weight within the intervention group from baseline at 6 months in this study. Similarly, there was a significant change in BMI in the intervention group than in the control group, which meant a more accurate weight loss representation. This result is consistent with previous studies that reported effects on weight loss in patients with NAFLD [17,29]. Weight loss is recommended for the treatment of NAFLD in the absence of pharmacological treatment. Previous intervention studies on patients with NAFLD have mainly focused on improving physiological outcomes, including body weight and clinical aspects [17,29,30].

In this study, other physiological outcomes, such as liver fat score, AST, ALT, and ɣ-GT levels, significantly changed in the intervention group at 6 months. The previous studies presented that weight loss through lifestyle modification is known to be associated with a reduction in liver fat and recommend a 5% minimum weight loss [5,31,32]. Lifestyle modifications, such as diet changes and exercise routines, have been reported to be effective in reducing body weight [33-35] but did not directly result in the reduction of participants’ liver fat scores in this study. The reason for this ineffectiveness in improving liver fat scores in this intervention may be related to the fact that participants did not present a significant body weight loss that could result in the improvement of NAFLD-related outcomes. Despite the lack of liver fat reduction at 6 months, there were significant changes in liver enzymes (AST, ALT, and ɣ-GT). Generally, liver enzyme levels in simple steatosis cases are mostly not high because their abnormalities are related to inflammatory processes [36]. Nevertheless, our results showed a reduction in the mean levels of liver enzymes at 6 months. Furthermore, we found that changes in liver enzymes were significantly different only among participants with a high level of intervention compliance. Although AST and ALT have a limitation of moderate accuracy in evaluating the fatty liver [37], this result suggests that further intervention studies need to be developed to identify the effects of changes in liver enzymes and to determine the significant level of weight loss and adherence on these changes.

In the interest of improving the health conditions of patients with NAFLD, psychological factors should also be taken into consideration, as many studies have reported a relationship between physiological biomarkers and psychosocial factors [38,39]. In this respect, our study identified that participants’ level of self-management improved in the intervention group compared to the control group and that the SMART-Liver app was significantly effective in improving self-management among patients with NAFLD at 6 months. Self-management behaviors that modify unhealthy lifestyles related to diet and lack of physical activity are a cornerstone of NAFLD treatment [35]. Several studies have conducted lifestyle modification interventions using mobile phones or other electronic devices and have identified the effects of physiological factors in patients with NAFLD [17,29,40,41]. However, other interventions targeting patients with chronic illnesses have evaluated their effects on psychosocial variables, such as self-management, self-efficacy, and quality of life, as well as on physical factors [40-43]. Our findings support the idea that lifestyle modification interventions can significantly improve psychosocial and physical parameters among patients with NAFLD.

Even though NAFLD is an asymptomatic disease, the SMART-Liver program was also effective in managing NAFLD’s nonspecific symptoms, such as fatigue. Recently, Golubeva et al [44] reported that fatigue was almost twice more common in patients with NAFLD than in those without it. However, chronic liver disease fatigue management remains a challenge because the pathophysiology of fatigue is complex. From this perspective, health outcomes following lifestyle modifications such as weight loss or reduction in liver fat may positively affect fatigue.

Additionally, participants with high compliance (90% and above) showed higher reductions in body weight and BMI at 6 months. Clinical factors (liver fat, AST, and ɣ-GT) and psychosocial factors (self-management and self-management knowledge) were also significantly improved by higher participant compliance (90% and above) compared to other groups. Similarly, there were significant differences in the level of fatigue and quality of life among compliance groups, with higher changes in the 70% or more compliance groups than in the group of 70% or less. Generally, higher compliance can improve health outcomes [45-47]. However, evidence on the standard level of compliance in lifestyle modification interventions using mobile apps is lacking, and many studies that included patients with various chronic diseases have mainly reported medication adherence [48,49]. Our study was conducted to derive changes in health outcomes according to lifestyle behavior modification compliance. These findings may be helpful in setting goals to improve the outcomes of the intervention in this population.

In this study, the attrition rate in the intervention group was low (9/111, 8.1%). The attrition of mobile-using interventions for chronic diseases was previously reported to be 40% [49]. In prior interventions in patients with NAFLD, attrition rates ranged from 5% to 27%, and interventions using the coaching method had especially lower rates [15,16]. Health coaching is an individual education method that can improve health behaviors and the effectiveness of interventions for chronic disease management [50]. Our study applied the coaching method to guide tailored goals by considering the participants’ circumstances. The participants were provided counseling via a remote meeting system halfway through the intervention period, which reset the goal based on their behavior and compliance and discussed their obstacles in changing unhealthy habits. In addition, the number of male participants with NAFLD was higher than female participants in this study even though we did not control for the sex ratio. Generally, in research, the proportion of female participants is higher than that of male participants [51]. As this study did not analyze the difference between male and female participants, further research is needed to compare this aspect.

In addition, compared to other liver diseases, most patients with NAFLD have less severe physical symptoms, and they can continue to engage in social activities, including work, relatively without constraints. In this respect, patients could participate in this program using a mobile app without time or space constraints, and if they had obstacles in sustaining their healthy lifestyles, they could contact and receive help from our research team. This might contribute to lowering the attrition rate. Further studies in this population may be successful if conducted considering these individual approaches.

### Strengths and Limitations

We found that this lifestyle coaching intervention using a mobile app was successful in improving the level of self-management, which could facilitate healthy behaviors for weight loss and reduction of liver fat. Therefore, the results of this study can help future studies to develop lifestyle modification interventions and apply them to interventions for patients with other chronic diseases.

This study had some limitations. First, the intervention was conducted in a single hospital with only mobile phone users using the Android system. Although the use of mobile phones has increased, it can be affected by age, educational level, and household income. Therefore, future studies could include various types of mobile phones to diversify the target population. Second, the intervention involved counseling for goal setting via a remote meeting system that was not embedded in the app. The research team separately provided a meeting link to each participant, even though the meeting was informed through a chat channel within the app. Future studies may benefit from the development of an app with all possible functions needed for intervention. Finally, it would be beneficial to identify the effects of self-management on participant groups classified by NAFLD progression or clinical findings. Additionally, we did not collect data on app usage of frequency in this study. However, this frequency may be associated with the intervention effect [52]. Further study may be conducted on the relationship between intervention effect and app usage frequency in this population.

### Conclusions

We developed a mobile app–based intervention for patients with NAFLD that provides personalized information, counseling, and interactions to facilitate self-management behaviors. This study showed that mobile app–based lifestyle modification interventions for patients with NAFLD effectively improved their physiological and psychosocial health outcomes. Our results suggest that future studies should include a more diverse and wider population and evaluate the effects of psychosocial aspects on self-management, as well as on clinical health outcomes in lifestyle coaching interventions using a mobile app. To confirm the effectiveness of the only coaching intervention, further studies that consider a mobile app–based lifestyle intervention without coaching as a group should be conducted.

## Appendix

Multimedia Appendix 1 CONSORT-EHEALTH checklist.

Multimedia Appendix 2 Pattern of mean changes in outcomes for 6 months in the intervention group.

## Abbreviations

ADDIE: Analysis, Design, Development, Implementation, and Evaluation
ALT: alanine transferase
AST: aspartate aminotransferase
NAFLD: nonalcoholic fatty liver disease
ɣ-GT: gamma-glutamyl transferase

## Data Availability

The data sets generated and analyzed during this study are available from the corresponding author on reasonable request.
