# Correction: Mobile App–Based Lifestyle Coaching Intervention for Patients With Nonalcoholic Fatty Liver Disease: Randomized Controlled Trial

**DOI:** 10.2196/57499

**Published:** 2024-02-27

**Authors:** Oh Young Kwon, Mi Kyung Lee, Hye Won Lee, Hyerang Kim, Jae Seung Lee, Yeonsoo Jang

**Affiliations:** 1 College of Nursing, Brain Korea 21 FOUR Project Yonsei University Seoul Republic of Korea; 2 College of Nursing, Mo-Im Kim Nursing Research Institute Yonsei University Seoul Republic of Korea; 3 Frontier Research Institute of Convergence Sports Science Yonsei University Seoul Republic of Korea; 4 Department of Internal Medicine, College of Medicine Yonsei University Seoul Republic of Korea; 5 Yonsei Liver Center Severance Hospital Seoul Republic of Korea; 6 Department of Nursing Science VISION College of Jeonju Jeollabuk-Do Republic of Korea

In “Mobile App–Based Lifestyle Coaching Intervention for Patients With Nonalcoholic Fatty Liver Disease: Randomized Controlled Trial” (J Med Internet Res 2024;26:e49839) the authors made two corrections.

In the first box of Figure 2, the following phrase:

Assessed for eligibility

Has been replaced with the following, as visible in the attached figure:

Assessed for eligibility (n=138)

Furthermore, the Acknowledgments section has been changed from:

This research was supported by the Brain Korea 21 FOUR project funded by the National Research Foundation of Korea, Yonsei University College of Nursing. We would like to thank Doing Lap for their cooperation and help throughout this research with their special technology. During the preparation of this, work the authors did not use generative artificial intelligence in any portion of the manuscript writing.

And will now read as follows:

This research was supported by the Basic Science Research Program through the National Research Foundation of Korea funded by the Ministry of Education (2017R1D1A1B04032264) and the Brain Korea 21 FOUR project funded by the National Research Foundation (NRF) of Korea, Yonsei University College of Nursing. We would like to thank Doing Lap for their cooperation and help throughout this research with their special technology. During the preparation of this, work the authors did not use generative artificial intelligence in any portion of the manuscript writing.

The corrections will appear in the online version of the paper on the JMIR Publications website on February 27, 2024, together with the publication of this correction notice. Because this was made after submission to PubMed, PubMed Central, and other full-text repositories, the corrected article has also been resubmitted to those repositories.

**Figure 2:**
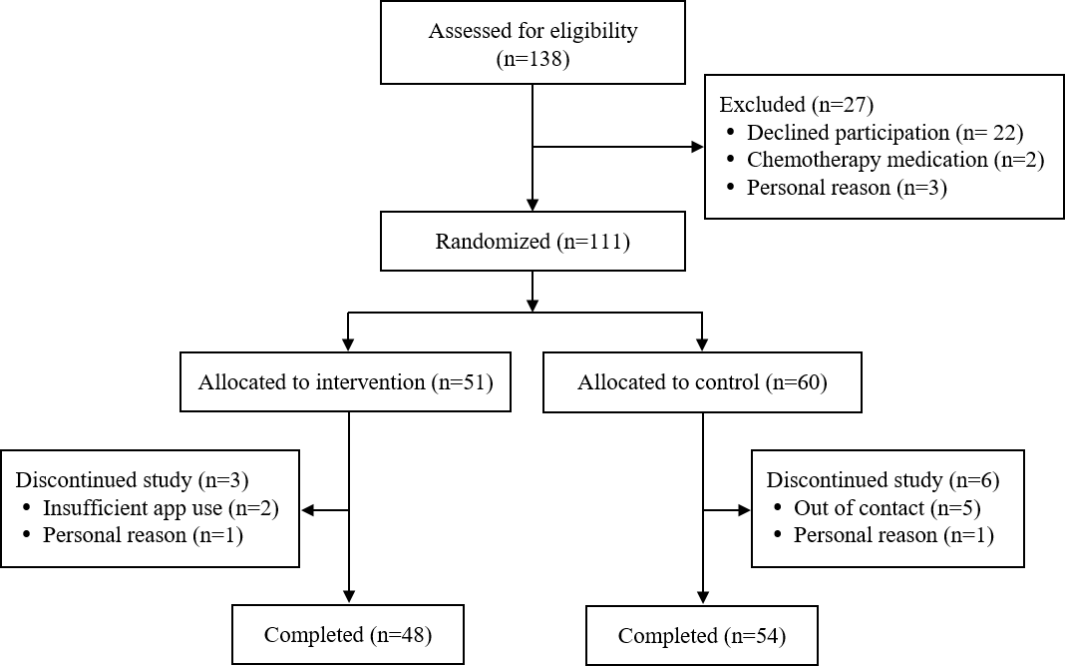
Summary of participation flow diagram.

