# Supplementary figures and images for: Mobile App–Based Lifestyle Coaching Intervention for Patients With Nonalcoholic Fatty Liver Disease: Randomized Controlled Trial
Source: J Med Internet Res. 2024 Feb 15;26:e49839. doi: 10.2196/49839 (PMC10905353; doi:10.2196/49839)

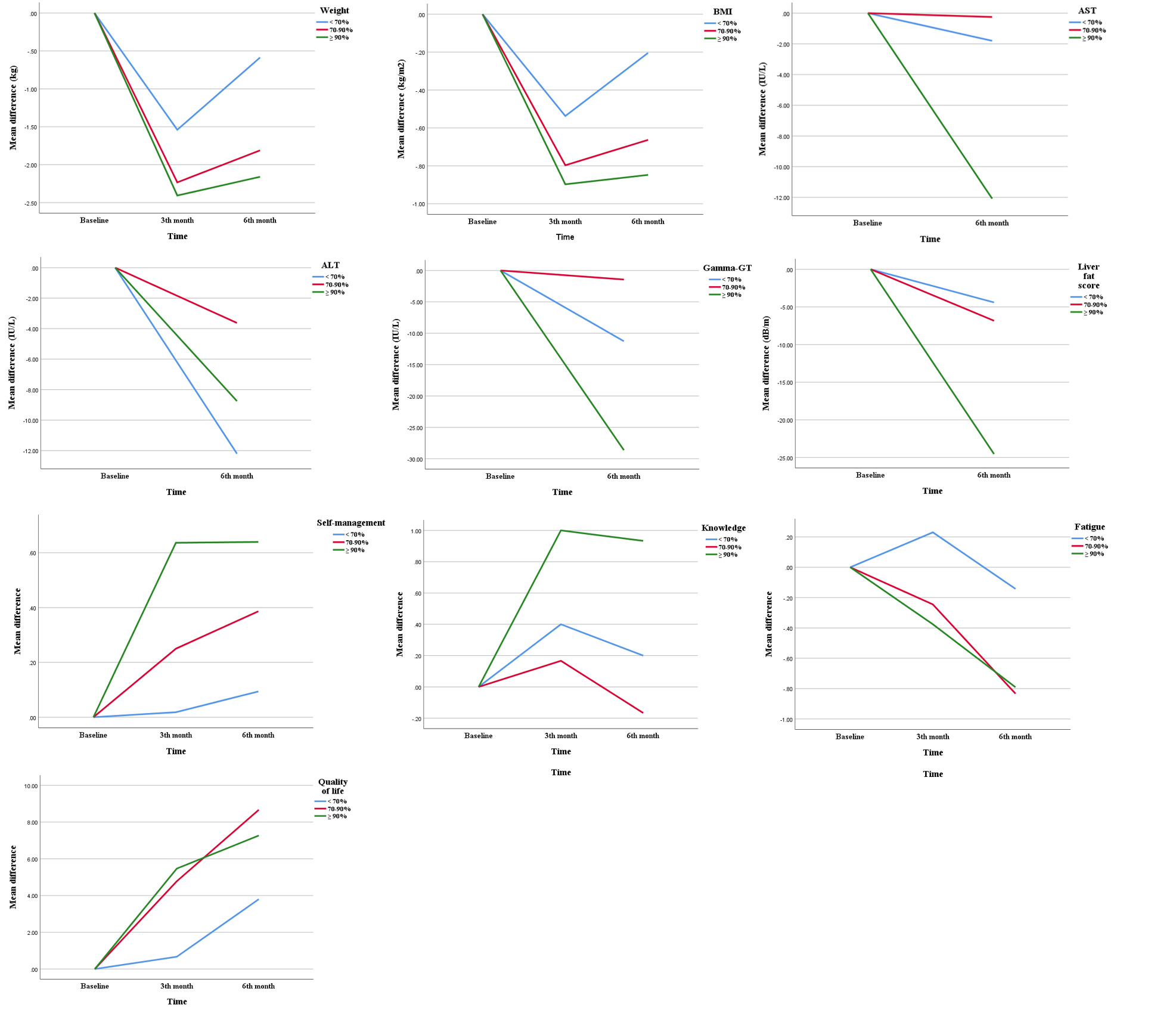

Supplement: Multimedia Appendix 2 [file jmir_v26i1e49839_app2.png]
